# Assessing geometric microliths as cultural markers through an analysis of shape variation and projectile performance

**DOI:** 10.1038/s41598-025-95054-2

**Published:** 2025-04-02

**Authors:** Alfredo Cortell-Nicolau, Alastair Key, Antoni Palomo

**Affiliations:** 1 Max Planck Institute for Evolutionary Anthropology, Deutscher Pl. 6, 04103 Leipzig, Germany; 2 Department of Archaeology, University of Cambridge, Cambridge, CB2 3DZ UK; 3 Department of Prehistory, Universitat Autònoma de Barcelona, Edifici B, C/Fortuna, 08193 Bellaterra, Barcelona Spain

**Keywords:** Projectile penetration potential, Force, Energy, Geometric microliths, Experimental archaeology, Mesolithic, Neolithic, Archaeology, Human behaviour

## Abstract

European geometric microlith shape variation is often used as a marker of cultural differences between groups of Mesolithic hunter gatherers and/or Neolithic farmers. Indeed, the 2D plan-view shape of these lithics is known to vary in spatially and temporally systematic ways between archaeological sites. Such differences are well evidenced in the Iberian Peninsula between the 9th and 8th millennia BP. Here we test an alternative hypothesis for the structured variation observed in geometric microliths: whether their plan-view shape significantly impacts the force, energy and displacement experienced when they are used as projectile tips. If functional differences between groups help to explain the shape variation observed in the archaeological record, then any role for cultural (non-functional social) explanations is potentially reduced. We undertook controlled static penetration tests using an Instron materials tester and an assemblage of replicated Iberian geometric microliths hafted to standardised wooden shafts. Results indicate that the maximum force required, energy used, and displacement at maximum force experienced by these hafted geometric microliths when used as projectile armatures is not significantly influenced by their 2D plan-view shape. Rather, gross form attributes such as maximum thickness, distance from the tip of the microlith to the start of the shaft, and the maximum width of the hafting substrate/adhesive are the greatest determinants of penetration ease, along with the positioning of the microlith when hafted. Our data therefore supports past research that proposes a cultural role for geometric microlithic shape variation in the European Mesolithic and Early Neolithic. Moreover, it highlights the functional importance of maintaining relatively thin microblades during microlith production, along with taking care to minimise the size of hafting components and the necessity to haft them in the most efficient way.

## Introduction

Geometric microliths are a specific type of lithic artefact most often inferred to have been used as part of composite arrowheads. They are usually trapezoid, triangular or lunate in shape. Their spatial and chronological distribution arguably dates from as early as 70,000 years before present (BP) in Africa^1,2^, but some of the clearest early examples are found in Asia and date to older than 30,000 BP^3–5^. In Europe, although some have been reported from the Solutrean period around 20,000 BP^6^, geometric microliths did not become predominant until the beginning of the Holocene. Indeed, from around 10,000 BP they slowly acquire omnipresence in the European archaeological record and are a dominant lithic artefact until the Early Neolithic, with reappearances throughout the Middle and Late Neolithic^7^. During this time in Europe, they show great size and shape variability, from (broadly speaking) Sauveterrian micro-triangles and micro-segments through to Castelnovian trapezes or larger Early Neolithic tranchettes. Often, an assemblage shows a wide array of intermixed forms which are commonly collated as part of named typologies. Due to this variation, they have often been considered one of the main cultural markers for the societies using them, and have been used to not only build periodisations for the groups involved—alone or in combination with other lithic proxies e.g.^8–12^—but also to help understand other relevant categorizations and patterns such as, among other things, social networks^13^, stratigraphic sequences^14^ or aggregation sites^15^.

Hindering their use as a cultural proxy is the fact that morphological differences observed between and within assemblages may indeed represent variable past cultural identities, or alternatively, they may represent differences in the use of these composite tools. Although several studies have assessed the potential functional applications of geometric microliths, principally through the use of experimental reproductions coupled with studies of breakage patterns^16–21^ and microwear analysis^22–27^, there remains an absence of work investigating how variation in their form influences their performance *during* a given task. That is, the potential utility of these lithic objects has been investigated and arguably demonstrated, but how utilitarian performance and any associated performance attributes relate to variation in their form remains unknown. This has major implications for studies that treat form variation in geometric microliths (on a stand-alone basis, or integrated within broader lithic assemblages) as a cultural proxy e.g.^11,21,28–31^. That is, despite their use as arrowheads seemingly being consistently supported see references above, but also^22,32^, their morphology—and any morphological differences observed within and between microlith assemblages—could be derived from variation in, or changes to, the use of these arrowheads within tasks or when different task-outcomes were intended; for example, hunting ground nesting birds relative to boars, or killing a rabbit relative to stunning it. If microlith morphometric variation was tightly linked to measures of utilitarian performance, and potentially therefore different functional tasks^34^, then the shape of these arrowheads could not reliably be used as a proxy for cultural diversity. Instead, they may better represent the ecology, and in turn faunal elements, present at the time and place of the artefacts deposition into the archaeological record. Such a scenario would reduce the social (cultural) explanatory power that has traditionally been attributed to the morphological variation observed in geometric microliths. Artefacts could have held a double value, even combining optimal functionality *and* optimal cultural value, but we adopt here a dichotomic framework to aid discussions on, and interpretation of, the relative role of each.

The style versus function debate of the 1980s and 1990s is highly pertinent e.g.^35–44^, as is more recent work built on these earlier ideas e.g.^45–49^. We do not intend to reproduce or exhaustively cover the full debate, however, some comments on this topic are useful. First, regardless of how one defines stylistic or functional traits (for debates on stochastic stylistic variation see Hurt & Ratika^44^), the cultural evolutionary mechanisms impacting each are governed by diverse selective pressures and, therefore, analytical approaches investigating each need to be tailored to one or the other. If the shape of any microlith artefact was conditioned by functional pressures, for example, then understanding the relevant selective environment (e.g. a specific type of prey or broader ecological niche, etc.) is essential to understanding why any variation (or lack thereof) exists. If traits are stylistic (even considering potential social functions sensu Sacket^37^), we can feasibly use geometric microliths as markers of past cultural groupings, and therefore gain insight into the social structure of these prehistoric populations, but given we cannot directly observe these past groups, the security of this inference is dependent on other explanatory factors (e.g., functional pressures) not playing a significant role.

To investigate the relative contribution of cultural drift, the founder effect and other cultural evolutionary mechanisms, including functional selective pressures, on past artefacts forms, researchers often rely on experiments. For example, it is possible to get a handle on the role of drift mechanisms through experiments that replicate the production and social transfer of past artefact forms e.g.^50,51^. In this scenario, if changes to the morphometry of geometric microliths were identified as being governed by social dynamics internal and specific to the group (of modern participants) who produced the artefacts, these objects could feasibly continue to aid our understanding of social chrono-spatial patterns in the European Mesolithic-to-Neolithic period. Similarly, experiments that investigate the potential role of functional selective pressures on artefact forms are also common e.g.^52–55^. In these investigations, experimentation allows for specific utilitarian hypotheses to be tested—including form-function relationships— within diverse conditions, with the internal and external validity of any results varying dependent on the specific experimental set-up^56^. Of most relevance here are studies that investigate potential performance characteristic differences between arrowheads or atlatl dart armature forms, with examples ranging from mathematical modelling^57^ and highly controlled machine-performed penetration tests^58–62^ through to those conducted by human participants using replica whole artefact-systems cf.^63^ to propel projectiles into simulated or real animal carcasses^17,21,64^.

Here, we investigate whether the form of geometric microliths can be confidently used as a proxy for cultural identity by experimentally investigating an alternative utilitarian hypothesis. Our approach relies on understanding whether microlithic morphometric variation has an effect on their performance (and in turn, inferred utilitarian efficiency) when hafted as arrowheads. If a form variable is demonstrated to have an effect on the performance, then it feasibly could have been a relevant functional factor for Mesolithic and Neolithic individuals. The rationale is that if shape does not alter microlith performance, then efficiency differences within ‘real world’ (i.e., prehistoric hunting) conditions would have been minimal and tool design choices would not have been linked to functional optimisation. We would like to clarify that our intention is not to understand which shapes may perform better than others but, rather, to assess whether shape affects performance at all. We do this through a combination of experimental replication, mechanical testing and statistical modelling. First, a replica assemblage of geometric microliths based on the shapes found in Eastern Iberia were produced with intentionally wide ranges of morphometric variability. Second, we undertake controlled static penetration tests following Sitton et al.^61^ and Mullen et al.^60^ to investigate form-performance relationships. Finally, we statistically assess which factors lead to optimal levels of performance when geometric microliths are used as projectiles.

## Methods and data

Ballistic analyses are fairly common in prehistoric archaeological research e.g.^20,62,64–70^. To the best of our knowledge, while limited-in-scale actualistic experiments investigating potential types of utility for geometric microliths have been published e.g.^17,18^, large-scale structured analyses exploring form optimisation and performance variation are lacking. For the present study, we focus on the performance of geometric microliths and hence it is useful to define the term ‘performance’ in advance. One way to think about performance is via the optimisation of an artefact’s production costs (e.g., time, energy, risk cf.^71^). Neeley and Barton^72^ provide clear time-costs during the production of geometric microliths, with each taking an average of 2–3 min (also see below), and this being, along with their modularity and ease of substitution, key for their success see^73^. Another relevant performance measure for microliths concerns the ability to track fauna see^74^. In theory, after an arrow strikes an animal microliths can detach from the haft and contribute to internal bleeding, more easily facilitating the tracking of the wounded prey. In this context, performance could be further enhanced by the potential use of poison e.g.^75–77^. These performance characteristics cf.^43^ were potentially important to the design of geometric microliths, but of principal concern here, and with most prehistoric projectile research see^78^, is the relative ability of the lithic armature to penetrate intended targets under low force and energy (incl. velocity) requirements. Indeed, if an arrowhead propelled at a given velocity cannot perforate an animal’s skin then its performance may be considered ineffective, if it perforates but only minimally penetrates performance would be considered inefficient, and if it both perforates and penetrates to a depth suitable enough to cause substantial injury performance can be considered efficient. Importantly, inefficient-to-efficient can be viewed as a scaled measure and recorded as such. Thus, in this work we specifically test the capacity of the geometric microliths to penetrate a substrate and how this relates to force and energy inputs, and we refer to performance as such.

According to these lines of thought, in order to assess whether geometric microlith morphometry/shape impacts efficiency we undertook three broad work packages, as follows.

### Replication and geometric morphometric process

First, an experimental sample of 29 geometric microliths were created, based on 14 defined shapes found during the Iberian Late Mesolithic and Early Neolithic^9,11,15,79^. It was our intention to replicate most of the typologies found in the area during this time, but we acknowledge outliers may exist in the archaeological record.

**Fig. 1:**
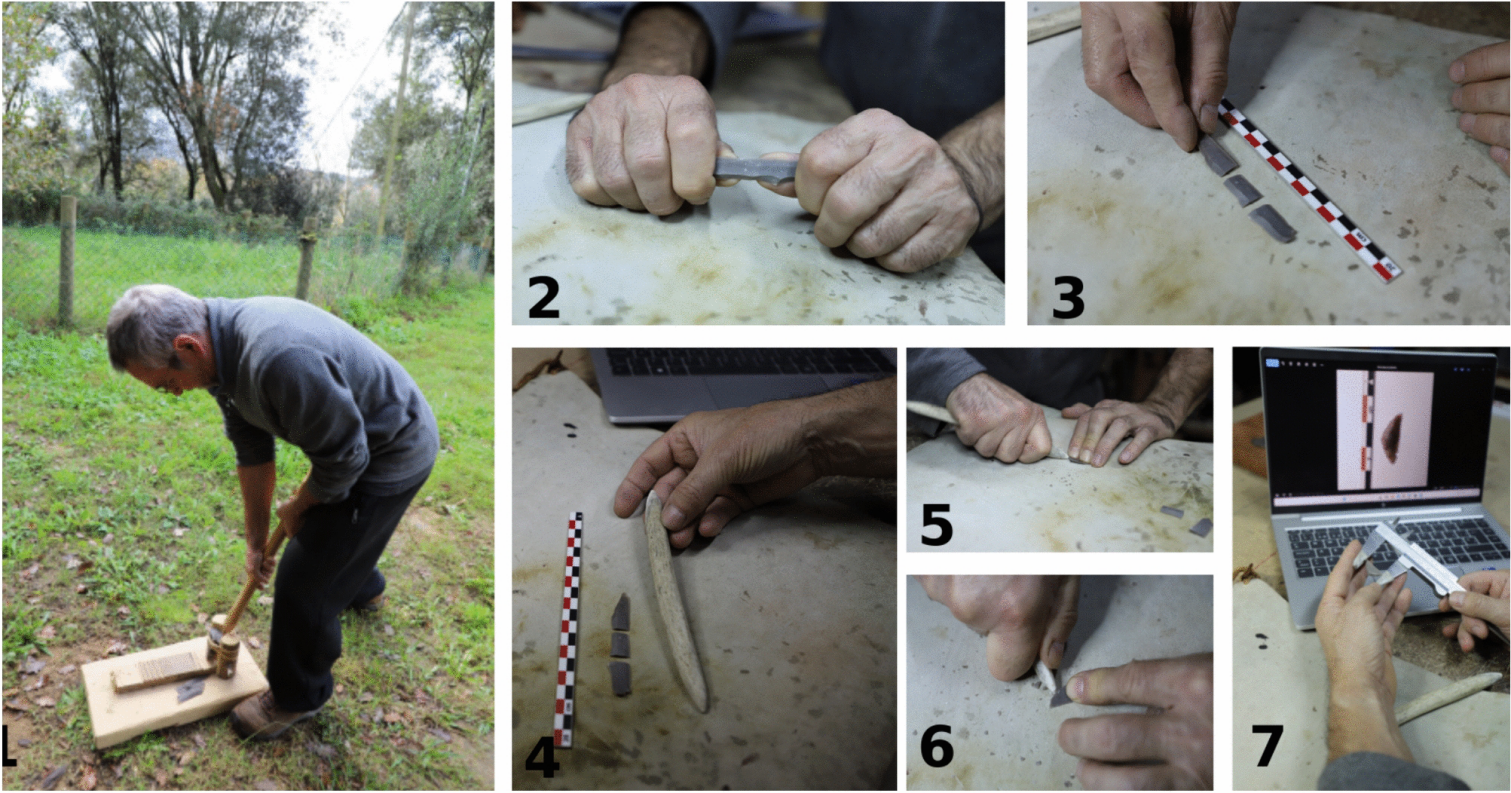
Experimental production process of the geometric microliths. 1—Production of blades through abdominal pressure; 2—Blade fracture through bending; 3—Blade fragments obtained through bending; 4—Blade fragments and deer antler tool used for shaping the geometric; 5, 6—Pressure retouch; 7—Comparison of the experimental versus the archaeological geometric. The individual in the image has given consent for publication in an online open-access journal.

Each microlith was produced from small blades knapped through abdominal pressure by AP, following the experimental process shown in Fig. 1. Fine grained chert gathered from Normandy (France) and Aragon (Spain) was used, and our choice for blade selection was guided by the intended final geometric shape. Based on prior work the two highly-siliceous and homogenous raw materials likely display similar sharpness measures^80^. In general, the replica blades presented transversal trapezoidal sections and longitudinal straight sections with slightly convex distal sides. All geometrics were segmented through flexion of the blade. Slim blades aid the segmentation process by allowing greater control over the fracture, with ‘clean’ fracture perpendicular to the long axis being more common. Once the blade was segmented we used a cervid antler tine with a sharp edge, along with a leather-protected table, to configure the geometric shape. Retouching (final shaping) typically did not last more than two or three minutes per geometric. Each geometric was hafted to the end of a 150 mm long wooden dowel, with the dowel used in place of an arrow shaft. We used a commercially available thermoplastic adhesive (hot-melt adhesive) to attach the lithics as this allowed the glue to be remelted, and in turn, each geometric could be used multiple times; in this case, in three distinct positions (see below). For the experiment, every point was positioned at the tip of the shaft. This was not their only potential prehistoric positioning and, in some specific cases, not even the most likely one^18,26,27^. We have proceeded this way because our intention was to assess the optimality of their penetrating capabilities, disregarding their lacerating properties. Each geometric was tested three times with three different positions on the tip of the shaft: obliquely pointing with the distal side, obliquely pointing with the proximal side and transversally/horizontally with the long base as potential cutting edge (see Fig. 4 below). For symmetric arrowheads we did the same for consistency, although we are aware that, in this case, there should be no significant differences between distal and proximal sides. This allowed us to investigate the impact of hafting position on penetration potential alongside the role of geometric shape/size variation. Regardless of their typology and morphometry, each geometric was hafted in the above mentioned three positions and using the same hafting method.

**Fig. 2:**
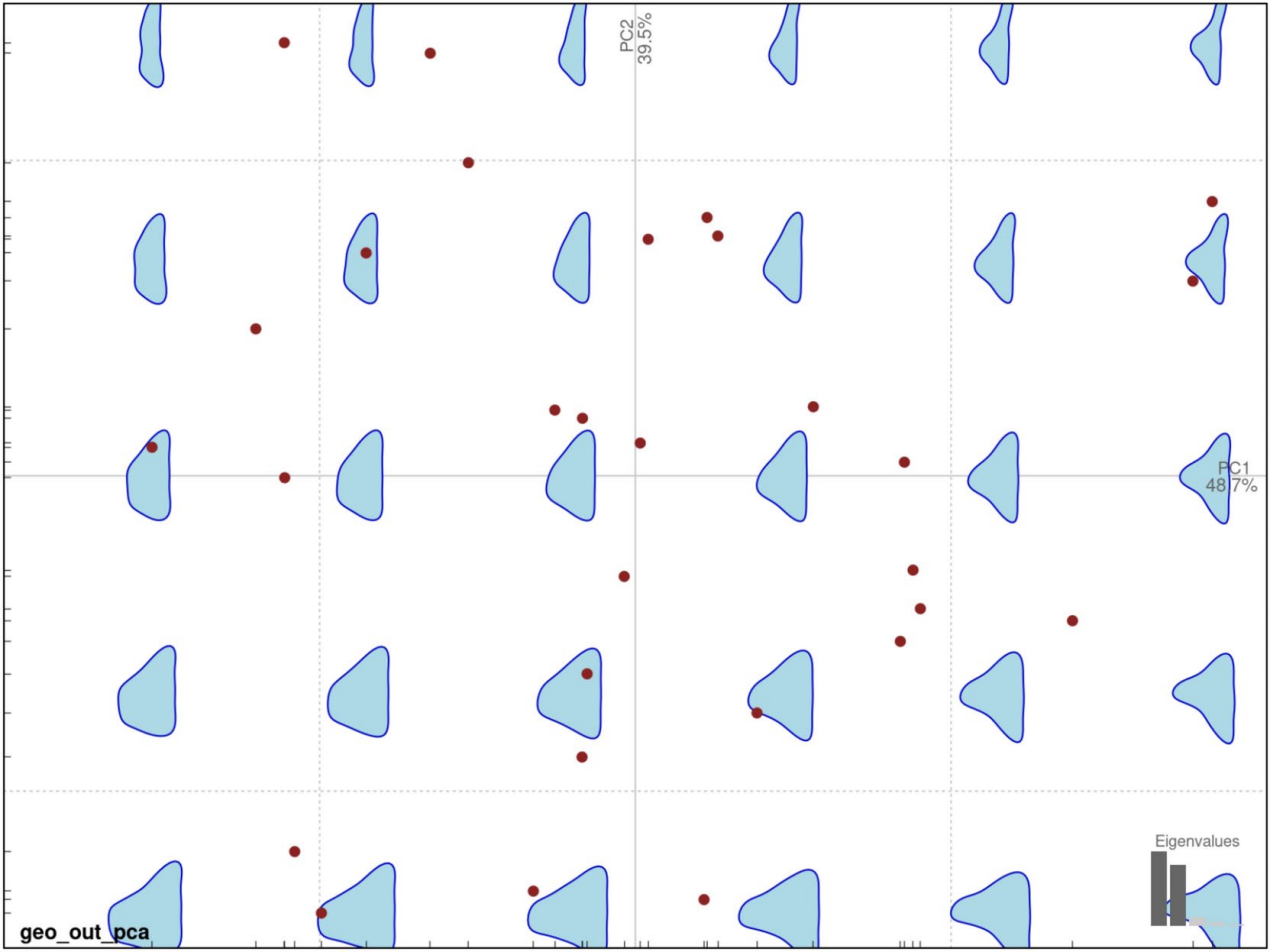
PCA on the geometric microliths studied. PC1 and PC2 have been included as co-variates to define the shape of the geometric microliths in the final model.

The shape of the geometric microliths was recorded using outline-based Geometric Morphometric Analysis (GMM), a technique widely utilised in modern archaeological practice. Details on the application of the method (outline or landmark-based) on lithic assemblages can be found elsewhere e.g.^15,30,81–90^. We first performed a General Procrustes Analysis (GPA) followed by a Fourier analysis with seven harmonics and principal component analysis (PCA). The intention here was not to use GMM as an end goal, as is typically the case but, rather, to use it as a proxy for recording each geometric’s morphology. With a cumulative variance of 88.2% for the first two components in our PCA (Fig. 2), we can interpret these components as broadly capturing the shape of each geometric. Therefore, instead of using linear measures to account for aspects of form variation, we use PC1 and PC2 (*M1* and *M2* respectively, see below) to describe the general outline shape of each microlith.

### Mechanical tests

For the second part of our analysis we investigated the penetration potential of each geometric microlith in each of its three hafted positions. We follow Sitton et al.^61^ and Mullen et al.^60^ and used an Instron materials tester (34SC-5 model) to perform machine-controlled static penetration tests. We secured the shaft of each hafted geometric into an upper grip on the Instron before lowering it at a controlled rate (50 mm/min) into a substrate. Throughout the force (newtons [N]), energy (joules [J]) and displacement (mm) experienced by the upper grip could be recorded, allowing us to observe how each metric varied depending on the replica lithic and hafting position used. Our specific interest was in recording peak force experienced (N), total energy (J) used, and displacement at the point of maximum force (mm), when pushing the microlith into the substrate. Each unique lithic and position combination was ‘thrown’ (i.e., pushed by the Instron into the substrate) three times, resulting in a total of 261 individual penetration tests (three times per geometric and hafting position) being conducted.

**Fig. 3:**
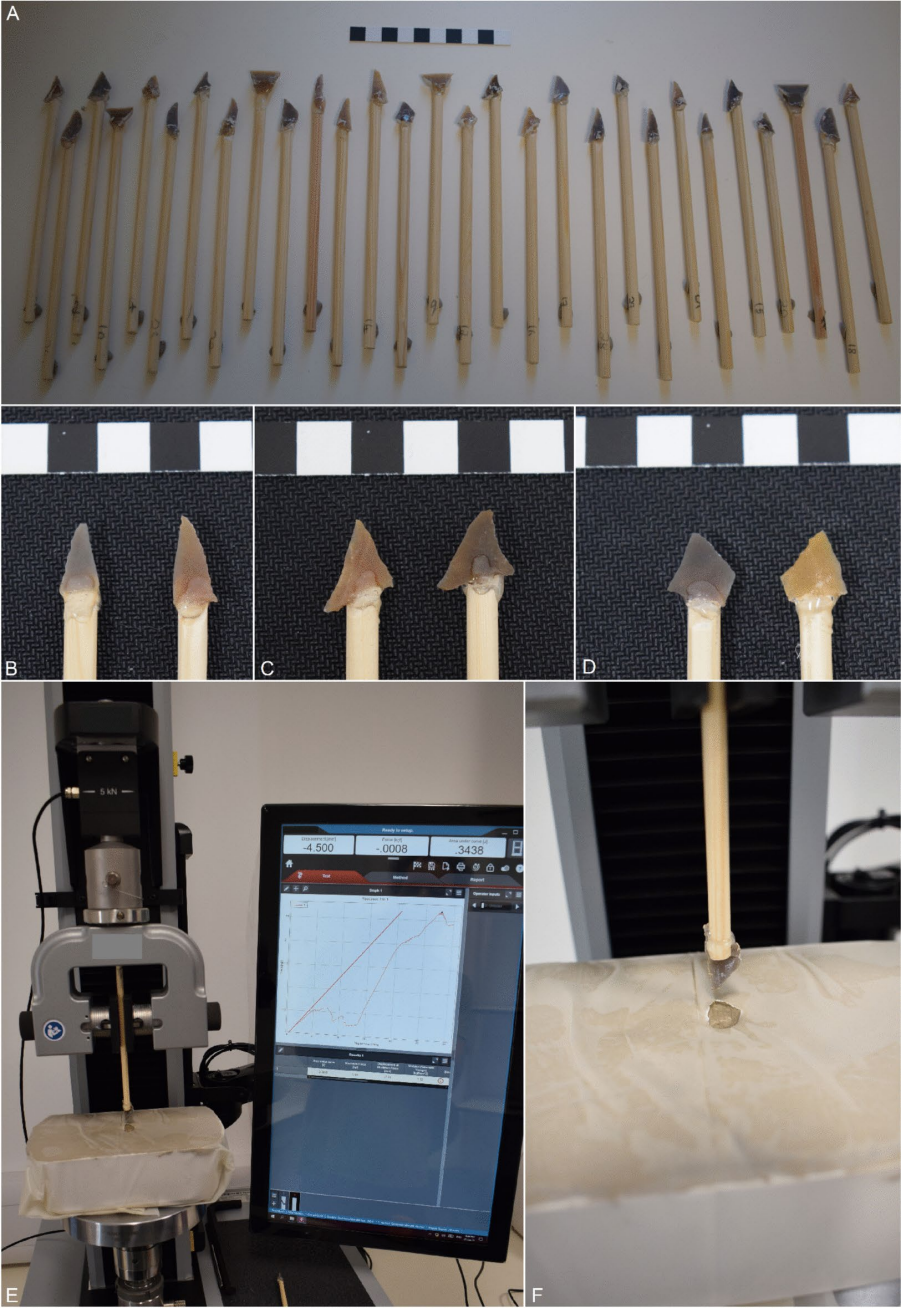
The sample of replica geometric microliths hafted to the wooden dowels (**a**), three pairs of geometrics prior to testing (**b**–**d**), the Instron device and wider experimental set-up, including an exemplar force–deformation curve (**e**), and a close-up of the experiment post-test (**f**).

It was essential for us to use an industrially produced material as the ‘target substrate’ to ensure testing conditions were identical for all samples. Following a large number of prior projectile experiments^59,61,91,92^ we used potters clay (terracotta [purchased from HobbyCraft in Cambridge, UK]) in place of meat, as the former has previously been demonstrated to display a reasonable proxy for the latter when investigating projectile penetration energy and forces in small armatures, although Key et al.^93^ stress the two materials are not directly interchangeable and differences increase when assessing larger armatures (which is not the case here). The clay was secured within a 10 × 15 cm plastic box, with the central portion of the clay being aligned underneath the microlith. On the superior surface of the clay we positioned a taut sheet of thin polythene plastic to act as a ‘skin’ that may at first deform and be pushed into the clay under pressure from the microlith, before being perforated after five mm of deformation and the lithic continuing into clay. Prior to the test beginning all lithics were aligned with the surface of the polythene such that they were touching but no force was recorded by the Instron. The lithics were pushed into the clay to a depth of 30 mm before the test ceased. Data were collected at a rate of 20 points/second with the maximum force (N) experienced, the displacement (distance moved) at the point of maximum force (mm), and the area (energy [J]) beneath the resulting force–displacement curve being collected for analysis (Fig. 3).

### Analytical framework

#### Variables and observations

We recorded the following three response variables:

- *E* (Energy): Total energy used by the geometric during the process of perforating the target and penetrating to a depth of 30 mm (measured in Joules).
- *F* (Maximum Force): The peak force experienced by the geometric when perforating the target (measured in Newtons).
- *D* (Displacement at Maximum Force): Displacement produced on the target by the penetrating arrowhead (measured in millimetres).

We considered a total of 10 potential predictors, as follows:

- *Sym* (Symmetry): Whether the gross shape of the geometric can be considered symmetric or not. Binomial.
- *I* (Inclination): Position of the geometric on the haft. Categorical with four categories: oblique distal, oblique proximal, horizontal, symmetric.
- *B* (Breakage): Whether the geometric breaks during the penetration testing. Binomial.
- *S.par* (Parallel width shaft): Width of the shaft measured parallel to the geometric’s positioning. Continuous in mm.
- *S.per* (Perpendicular width shaft): Width of the shaft measured perpendicular to the geometric’s positioning. Continuous in mm.
- *W* (Width). The maximum width of the geometric after hafting. Continuous in mm.
- *W.S.* (Width before shaft). Width of the geometric prior to the start of the shaft. Continuous in mm.
- *T* (Distance from tip to shaft). Distance from the tip of the geometric to the start of the shaft. Continuous in mm.
- *M1* (Morphometry 1). Plan-view shape of the geometric. PC1 result after the GMM analysis explained above.
- *M2* (Morphometry 2). Plan-view shape of the geometric. PC2 result after the GMM analysis explained above.

From these, *Sym* and *B* were discarded prior to the start of the analyses. For *Sym* this was because the symmetry of the geometric should be accounted for by its plan-view shape. As for *B*, no geometrics broke during the testing, so this co-variate became uninformative. The rest of the predictors were considered within the model. The low number of observations available from our analysis limited the complexity of our systematic component. Thus, to improve the model’s efficiency and reduce its complexity, two variables were modified: the variable *I* was converted to binomial with only two categories (oblique/horizontal), while the variables *S.par* and *S.per* were merged into one variable, *S*, the mean of the two (as in $S_{i} = \frac{{S \cdot par_{i} + S \cdot per_{i} }}{2}for\, i = 1,2, \ldots ,n$ with $n$ being the number of observations) and accounting for the overall thickness of the shaft.

**Fig. 4:**
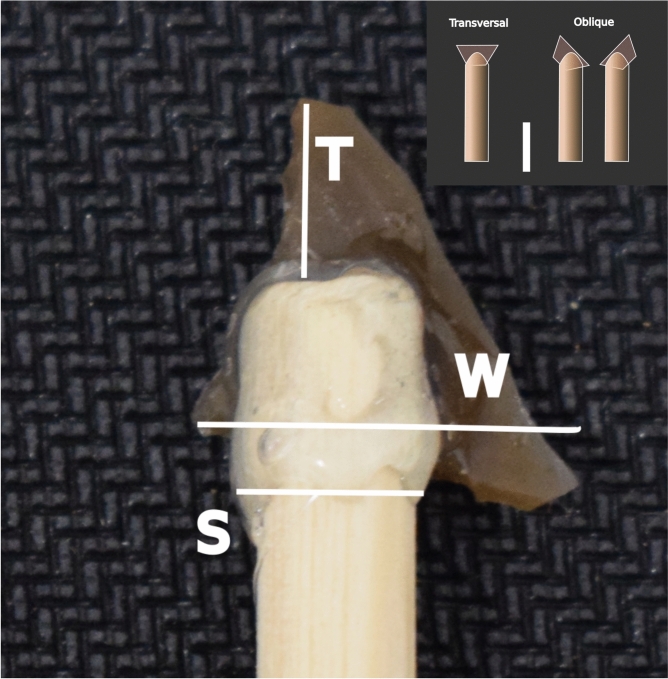
Predictors used as they were gathered for each geometric. The subimage in the top right hand corner shows the variable I and how each geometric was positioned along the shaft. For simplicity, the variable I was reduced to only two categories: transversal and oblique. M1 and M2 (not in this image) are explained in the previous section.

Our main criteria for co-variate inclusion in the final model was based on expert knowledge, usually advised over automatic and purely diagnostic procedures^94–97^. We complemented this with a collinearity analysis and a PCA on the rest of the co-variates to see whether they could provide insight for further model simplification, with no success. Thus, for the final systematic component of the model we included the co-variates *W*, *I*, *S*, *M1*, *M2* and *T*, as shown in Fig. 4. As it can be seen, each geometric was positioned in three different ways, although only two (oblique and transversal) were considered for the analysis. The decision to leave *W.S* out was also reinforced by the model’s performance worsening (higher WAICs) when it was included. To check for potential outliers, we computed the group Mahalanobis distance including the co-variates considered for the final model. Our results identified two potential group outliers for observations nine and 29. Observation nine had a negative impact on the model’s overall fitting, but observation 29 did not. Thus, we removed observation nine from the analysis, resulting in 86 observations for each response.

#### Models

We developed a hierarchical Bayesian model for each response (*E*, *D* and *F*) using R and nimble. The systematic component remained the same for each model and consisted of the variables mentioned above, plus one random effect group to account for the fact that each geometric was tested three times. All of our responses were strictly positive but they did not follow the same distribution, with some of them displaying skewness. To assess which specific type of model to use for the analysis of each response we tested a linear regression, linear regression with log-transformed data, gamma regression and lognormal regression. Our preferred model was chosen based on: (1) their standardised residuals and (2) their posterior predictive checks on mean and median, as adapted from Gelman^98,99^. According to this, we decided to use a standard linear regression for *E*, a lognormal regression for *F* and a gamma regression for *D*. We selected weakly informative priors consisting of (1) normal distributions with large standard deviation (100) for the parameters of the systematic component ($\beta_{1} , \ldots ,\beta_{n}$) and (2) half-cauchy distributions (coded as Student-t distributions with 1 degree of freedom) for the distribution’s parameters, such as the variances of the response and the random effects [see ^98,100^. Furthermore, we censored the response* D*. This is because the machine was set to a maximum level of 30 mm, and thus a concentration of values were observed on the upper limit of this range [29, 30], indicating that displacement at maximum force values for these samples could theoretically have exceeded 30 mm. Hence, we censored this response at 29.5, which returned a much better performance of this model.

**Fig. 5:**
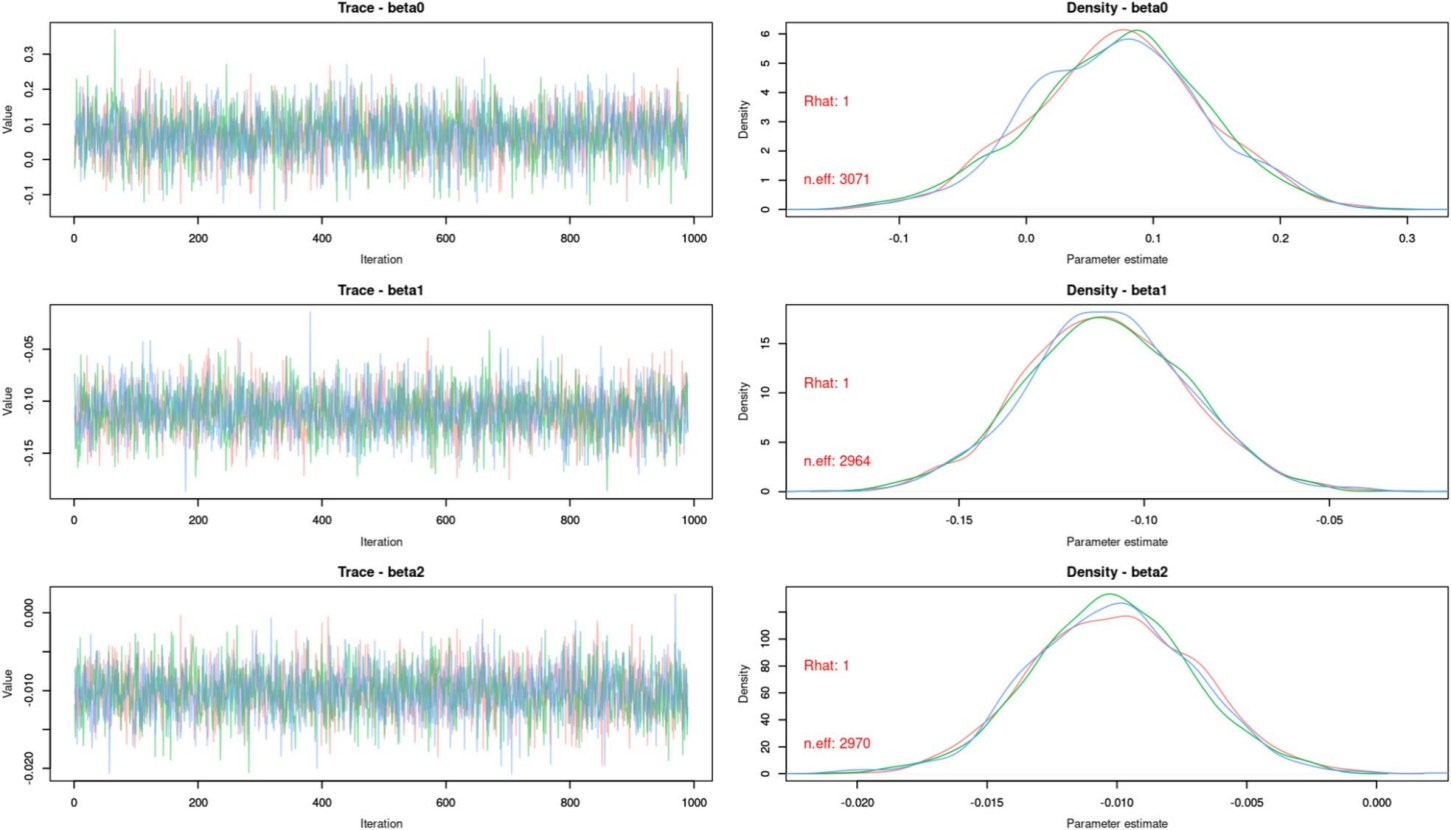
Convergence of the MCMCs. The pattern in the left hand side images, essentially random and without peaks, indicates good convergence. The same can be said for the right hand side images, which are approaching normal distributions, with Gelman-Rubin indices (rhat) = 1 and with number of effective sizes around 3000 (usually anything higher than 200 can be considered enough). Due to space constraints, only three parameters are shown. The rest can be observed in the code and plots provided as supplementary material.

We estimated the parameters using an MCMC with 300,000 iterations, burn-in period of 3000 and sampling every 300 iterations to obtain 1,000 non-autocorrelated values for each parameter’s posterior distribution. Chains converged properly (Fig. 5), with Gelman-Rubin equaling 1 for the vast majority of parameters (occasionally 1.01) and with number of effective sizes > 2000 in any case.

All data and code for the analyses mentioned above, along with exploratory analysis, plots and further model diagnostics are available in the supplementary material. Supplementary material has been prepared as a git repository, with its dedicated readme, and can be found at https://github.com/acortell3/Geo_performance. We have used the language R^101^ and the R packages nimble^102,103^, MCMCvis^104^, coda^105^, psych^106^ and Momocs^107^.

## Results

**Fig. 6:**
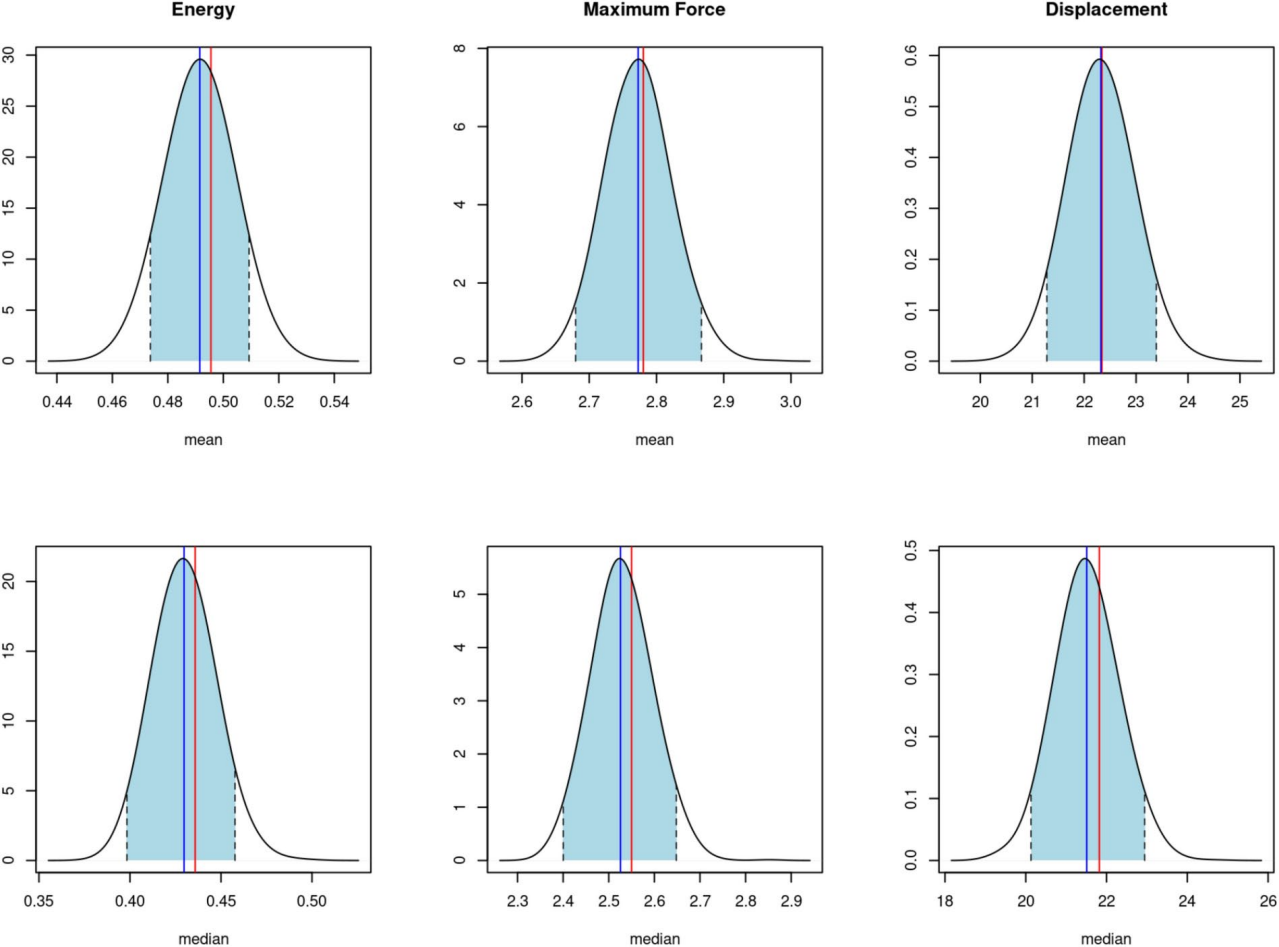
Posterior predictive checks on mean and medians of the models selected. Red is the observed mean/median and blue is the prediction from our parameters’ posterior distributions.

Our results focus on the statistical models developed to assess the effect of the different co-variates on the response variables. One of our main criteria when selecting the best model to use was whether it was capable of fully recapturing the mean and median through a posterior predictive check (Fig. 6) (see Gelman^98^, and see Crema & Shoda^108^ and Timpson et al.^109^ for archaeological applications). In this regard, the models selected obtained practically identical means (Table 1) with very similar medians (Table 2); these data act to confirm the models worked as expected.

**Table 1:** Posterior predictive checks on means.

	Area	Maximum force	Displacement
Observed	0.5	2.78	22.34
Predicted	0.49	2.77	22.32

**Table 2:** Posterior predictive checks on medians.

	Area	Maximum force	Displacement
Observed	0.44	2.55	21.82
Predicted	0.43	2.53	21.5

**Fig. 7:**
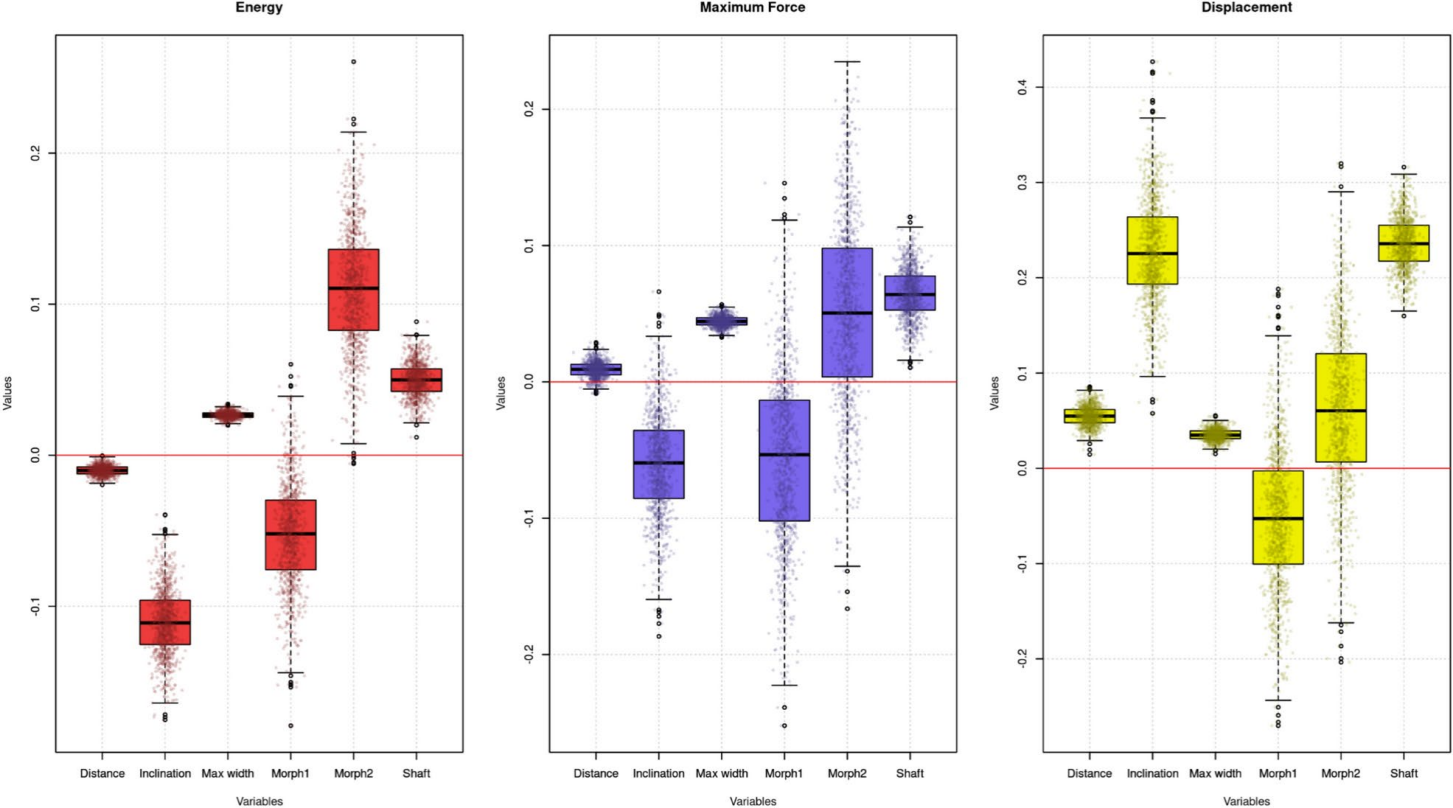
Posterior distributions of the parameters considered for each model (energy, maximum force and displacement). Effects can be considered relevant to the interpretation of geometric performance if their credible intervals deviate from 0.

After checking the adequacy of the model our main interest was to understand how the parameters considered affected the responses for each model (*E*, *D* and *F*). Figure 7 demonstrates some consistent trends. When analysed individually, *T* (the distance from the tip of the geometric microlith to the beginning of the shaft) has a small effect on the penetration of the arrowhead, considering the responses *E* and *D*. In the first case, as *T* increases, less force is required and, in the second, for higher values of *T*, more displacement would be recorded. The small values of these effects (see Table 3) make them almost neglectable. Taking into account the response* F*,* T* does not differentiate from zero. On the other hand, the inclination of the geometric microlith, *I*, plays a key role in penetration potential, with arrowheads obliquely positioned resulting in lower energy being required to penetrate the target and displacement at maximum force being greater. For these two responses, and as shown in Table 3, the posterior distributions for this parameter are significantly different from zero, showing its potential effect. Although the upper credible interval on this co-variate on the response *F* is slightly above zero, its lower credible interval and mean suggest that an arrowhead obliquely positioned would require reduced force to penetrate the target, but this is not strongly supported by our results.

**Table 3:** Means and credible intervals of the posterior distributions of the parameters studied for each response.

	E (mean)	E (CI)	F (mean)	F (CI)	D (mean)	D (CI)
T (Distance)	− 0.01	− 0.02, − 0.01	0.01	0, 0.02	0.05	0.04, 0.07
I (Inclination)	− 0.11	− 0.16, − 0.07	− 0.06	− 0.13, 0.01	0.23	0.13, 0.33
W (Maximum width)	0.03	0.02, 0.03	0.04	0.04, 0.05	0.04	0.02, 0.05
M1 (PC1)	− 0.05	− 0.13, 0.01	− 0.06	− 0.17, 0.06	− 0.05	− 0.2, 0.09
M2 (PC2)	0.11	0.04, 0.19	0.05	− 0.08, 0.18	0.06	− 0.09, 0.24
S (Shaft)	0.05	0.03, 0.07	0.06	0.03, 0.1	0.24	0.19, 0.29

The maximum width of the arrowhead (*W*) and the shaft/hafting width (*S*) also play a key role in the penetrating capabilities of geometric microlith arrowheads, in all cases clearly differentiating from zero. For the former, higher values of *W* indicated that the arrowhead requires greater force to perforate the target, greater energy during penetration and deeper penetration at the point of maximum force. For *S*, we observed similar relationships, albeit with larger credible intervals. These, following Table 3 and Fig. 7, could be interpreted as thicker shafts requiring more energy and force to penetrate the target, along with greater forces being required at greater penetration depths. The effect of displacement at maximum force (019–0.29) is significantly larger than energy (0.03–0.07) and maximum force (0.03–0.1). Interestingly, for the variables representing the shape of the arrowhead (*M1* and *M2*) neither diverged from zero (indicating that the shape or morphometry of the geometric does not influence the penetration of the throw), with in some cases (e.g. for responses *F* and *D*) zero being almost within one standard distribution from the posterior’s mean. For the case of *E* as a response, the credible interval of *M2* (which could be broadly representing slimmer arrowheads) did differ from zero, although some of its values were still negative. The exact impact of the different co-variates regarding each response has been summarised in Table 3.

In sum, these results demonstrate the position of the geometric (oblique vs. transversal; predictor *I*) to have the strongest effect on performance during each test, followed by the shaft’s width (*S*) and the maximum width of the geometric (*W*). The distance from the tip to the shaft (*T*) does also have an effect, but it is small and not for all responses. Finally, 2D plan-view shape (*M1* and *M2*) does not appear to improve or worsen the penetrating capabilities of geometric microliths.

## Discussion and conclusion

Our main objective was to understand whether morphometric variation in geometric microliths impacts their performance when used as projectile tips (armatures). This allows us to assess whether their prehistoric design (form) was likely conditioned by utilitarian optimisation, or alternatively, if it was governed by other factors, including cultural constraints. As reported in the results, our analysis suggests the main factors affecting their performance as projectiles are their positioning on the shaft (oblique or transverse), the maximum thickness of the shaft (when hafted, including adhesive), the maximum width of the geometric and, to a lesser extent, the distance of the geometric’s tip to the start of the shaft. Plan-view shape does not seem to play an important role.

These results are consistent with the fundamental mechanics of projectiles as they penetrate substrate^110^. Oblique positioning on the haft and the lower shaft and geometric thickness measures, all contribute to reducing the surface area of the projectile perpendicular to the target as it penetrates, reducing the resistance experienced. Further, transversal arrowhead positioning, along with thicker shaft and arrowheads, result in increased displacement prior to maximum force being recorded meaning that once the arrow penetrates, the work experienced by the projectile appears to increase as it pushes deeper into the substrate, rather than being experienced closer to the point of initial penetration.

Admittedly, this study has not considered other elements which might impact penetration potential, such as the varying use of bows, spears or atlatls. Given the poor preservation of wood from the European Mesolithic and Neolithic we do not know a great deal about the bows used at this time, but there are exceptions in water-logged environments (see Piqué et al.^111^, Bailey et al.^112^ and see Bertin et al.^113^ for non water-logged case), as well as experimental studies assessing the potential requirements of prehistoric bows^114,115^. There appear to be differences in the wood used for bows during the Mesolithic and Neolithic, with the earlier hunter-gatherers usually preferring elm (*Ulmus sp.*) and, with exceptions, farmers tending to use yew (*Taxus bacatta*). Archaeobotanical data in Northern Europe explains how yew expanded through Europe during the sixth millennium, which might explain how hunter-gatherers started to diversify their wood use around this time^116^. In any case, the technology for bow manufacture seems to be different between the two periods, although at present this difference does not seem to allow easy discrimination between potential different uses^33^.

Some other technological elements such as the bow’s size or the fletching of the arrow might also impact their performance^115,117^, but we understand even less about the selection of these factors in prehistoric Europe. Nevertheless, in our case it is important to consider that we are not analysing the overall hunting performance, which would refer to the full hunting artefact-system^63^, including the bow, but how the shape of the arrowhead improves this performance. Arrowhead shape has been reported to change depending on the method of hafting used in some contexts (see Bettinger & Eerkens^117^ for the case of Palaeoindian points). This is particularly relevant because it is known that microliths were not always located on the tip of the shaft, but could also be used modularly along the side of shafts to form barbs in a harpoon-like system^18,27,118,119^. We have not considered this hafting disposition as we focus on the penetration capabilities of the arrowheads alone. Alternative methodological approaches that display the ‘wound’ track of the projectile e.g.^60,119^ would be required to investigate laceration capabilities. Further, it would require assumptions as to which microliths should be positioned on the tip and which ones should be positioned along the shaft see^7,120^. While it is true that we might have used some microliths as *tip* arrowheads when their prehistoric position was more often as a *shaft* arrowhead, this does not affect our results. Indeed, we have theoretically assessed the tip-functionality of ‘*shaft’* armatures, with our data demonstrating that if shape is a determinant of which microliths are hafted to the side of shafts and used for laceration, it is likely because of their performance during laceration and not penetration (although more detailed exploration of how side and tip armatures impact performance together is required^121^).

Our results are distinct to similar experimental data returned for Clovis points, with 2D plan-view shape (i.e., size having been controlled for) having been demonstrated to significantly impact their penetration depth (and in turn, likely force and energy required over the first 30 mm of penetration)^54^. This difference likely stems from the overall size of the armatures, with microliths being substantially smaller and the relative mechanical impact of shape differences being subsequently reduced. We did not consider cross-sectional shape here, so it is difficult to assess how our data compare to studies that found a relationship between this parameter and penetration depth e.g.^65,121,122^, but our measure of maximum thickness—along with its significant impact on performance—is a highly related metric with likely similar results.

Our main question here is, all else being equal, does the shape of geometric microliths affect their capacity to be used as a projectile? Our results point to form-attributes other than microlith 2D plan-view shape, including hafting sections and gross size attributes (e.g., thickness), being more relevant to determining their penetration performance. This supports past research proposing that geometric microlith shape can be used to help understand cultural differences between groups of Mesolithic hunter gatherers and Neolithic farmers^11,15,28,29,124–126^. Indeed, our research suggests that their morphometry and shape could potentially be used to assess complex questions regarding group identity and cultural transmission patterns in Prehistory e.g.^126–130^. Moreover, due to their role as a distinctive functional end product (as opposed to, for example, scrapers, blades or microburins), we believe that geometric microlith shape may be one of the most informative well-preserved cultural markers of this time e.g.^9,11,79,131–135^. If and how geometric microlith shape was related to social functions, however, is another question altogether, and one beyond the scope of our experimental paper.

In sum, we have shown that the shape of these arrowheads does not appear to retain information relating to the penetration potential of projectiles, and instead they likely contain cultural information relevant to understanding the group dynamics and interactions of these past peoples. While additional studies are required to further support (or refute) our inferences, this work provides a novel utilitarian foundation from which to analyse geometric microlith morphometric variability across broad geographic and diachronic ranges.

## Data Availability

All the data and code generated for this article can be found in a reproducible format here https://github.com/acortell3/Geo_performance.
